# Versatile and Controlled Synthesis of Degradable, Water-Soluble Bottlebrush Polymers with Poly(disulfide) Backbones Derived from α-Lipoic Acid

**DOI:** 10.1021/acsmacrolett.4c00839

**Published:** 2025-02-03

**Authors:** Ivan O. Levkovsky, Lucca Trachsel, Hironobu Murata, Krzysztof Matyjaszewski

**Affiliations:** Department of Chemistry, Carnegie Mellon University, Pittsburgh, Pennsylvania 15213, United States

## Abstract

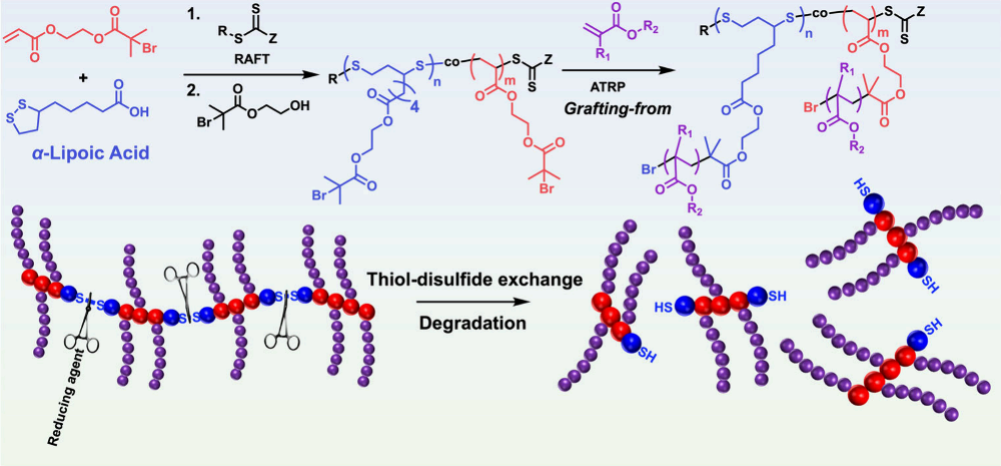

Bottlebrush (BB) polymers, with their densely grafted side chains and unique architecture, are highly advantageous for drug delivery due to their high functional group density for drug conjugation, unimolecular nature, and enhanced biodistribution properties. These attributes enable extended blood circulation half-life, improved tumor tissue penetration, and high tumoral drug accumulation. However, the typically nondegradable, all-carbon backbones of most BB polymers limit their suitability for applications requiring controlled clearance and biodegradability. To address this, we developed degradable BB polymers with poly(disulfide) backbones synthesized via reversible addition–fragmentation chain transfer (RAFT) copolymerization of α-lipoic acid (LA), a renewable and readily available compound, with acrylate-based inimers. These copolymers feature degradable backbones and initiating sites for subsequent BB synthesis. Using an atom transfer radical polymerization (ATRP) *grafting-from* methodology, we synthesized BB polymers with relatively low dispersities (*Đ* = 1.30–1.53), high backbone degrees of polymerization (*DP*_bb_), and high molar masses (*M*_n,MALS_ = 650–2700 kg/mol). The easily cleavable disulfide bonds enabled backbone degradation under mild reducing conditions. Beyond hydrophilic BB with tri(ethylene glycol) methyl ether acrylate (TEGA) side chains, we synthesized BB with cationic, anionic, and zwitterionic side chains, demonstrating broad monomer compatibility. This scalable approach produces water-soluble, degradable BB polymers with tunable architectures and predictable molecular weights. By addressing the need for degradability in BB polymers, this work advances their potential for drug delivery, offering enhanced functionality, biocompatibility, and sustainability.

Bottlebrush (BB) polymers consist of a polymer backbone with densely grafted polymer side chains that lead to the elongated bottlebrush structure.^1−4^ The steric repulsions and relatively short side chains give rise to several unique properties compared to other macromolecular architectures, namely low elastic moduli, high entanglement molecular weights, and tunable self-assembly behavior.^5−10^ In addition, biomedical applications of BB polymers have been investigated, as they exhibit good stability in solution, long blood circulation, effective tissue penetration, and the ability to carry many therapeutics per macromolecule if used for drug delivery.^11−16^ If the BBs were made biodegradable, this would further enhance their biocompatibility and ensure the clearance of the BB from the body after achieving their desired effect. Recently, there have been several reports on the synthesis of degradable BB polymers,^17−20^ with synthetic strategies commonly involving the incorporation of degradable repeat units into the BB main chain. These degradable units include silyl ethers,^17,18^ thioesters,^19^ and disulfides,^20^ which are labile in response to certain conditions or stimuli. However, there are several limitations to the degradable BBs reported thus far, most notably in achieving degradation under mild physiological conditions,^17^ low side chain density,^17−19^ low and uncontrolled degrees of polymerization (DP) of the backbone (*DP*_bb_) and low DP of the side chains (*DP*_sc_),^19,20^ tedious synthetic procedures,^17,19^ or limited versatility in side chain functionality.^18,20^ Notably, You et al. attempted to address these issues in a recent report on a highly controlled living ring-opening metathesis polymerization system that was extended to BBs, incorporating easily degradable 7-oxa-2,3-diaza-norbornenes into backbones.^18^ However, the BBs presented in this work still did not possess high *DP*_bb_, had low polymeric side chain density, and only one example of side chain composition (poly(ethylene glycol) methyl ether). Addressing all these issues enables the facile synthesis of BBs with precise architectures and controlled molecular weights while ensuring their degradability under mild conditions necessary for biocompatibility.

BBs with polymer backbones containing repeating disulfide units, or poly(disulfide)s, are particularly attractive for biological applications such as targeted anticancer drug delivery.^23,24^ Disulfide bonds are biodegradable and can undergo rapid thiol–disulfide exchange with glutathione (GSH), a physiological antioxidant present at especially high concentrations in tumor cells.^25−28^ A viable and frequently utilized approach to prepare poly(disulfide)s is through the ring-opening polymerization (ROP) of 1,2-dithiolane monomers.^29,30^ α-Lipoic acid (LA), a biologically derived and commercially available small molecule containing 1,2-dithiolane moiety, can undergo radical ROP to yield poly(disulfide)s.^31−33^ Recently, Hawker et al. reported the controlled synthesis of poly(disulfide)s through reversible addition–fragmentation chain transfer (RAFT) copolymerization of LA with acrylates (Figure 1A).^21,22^ The copolymerization of LA and *n*-butyl acrylate (BA) yielded polymers with controlled molecular weights and low dispersity (*Đ*). A kinetic study of the copolymerization revealed faster incorporation of LA compared to BA, favoring the formation of disulfide units in the copolymers. In addition, at 20 and 30% molar feed ratios of LA to BA, the final LA incorporation in the copolymers was 22 and 36%, respectively, which led to favorable degradation profiles of the copolymers with the mild disulfide-reducing agent tris(2-carboxyethyl) phosphine (TCEP). Verduzco et al. reported a *grafting-through* approach to synthesize degradable BBs with poly(disulfide) backbones by polymerizing an LA-based macromonomer through uncontrolled radical photopolymerization (Figure 1B).^20^ However, the resulting BBs exhibited uncontrolled molecular weights and small backbone DPs (*DP*_bb_ ≤ 35). Additionally, the backbone DPs were shorter than those of the grafted side chains (*DP*_sc_), resulting in architectures that resembled star-like polymers rather than typical BB polymers, which likely restricts the favorable properties associated with true BB polymers (Figure 1B).

**Figure 1:**
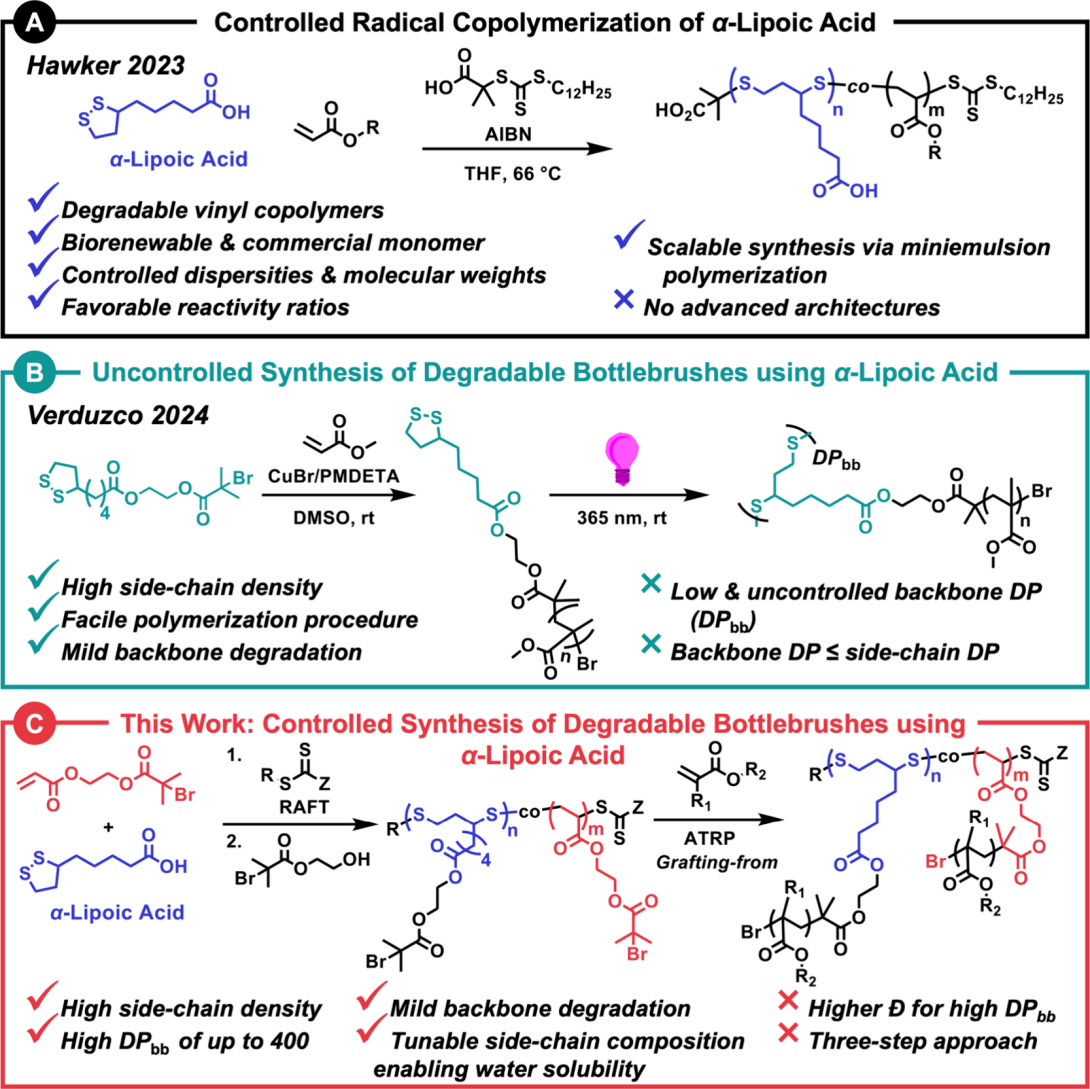
(A) RAFT copolymerization of LA and various acrylates to obtain degradable vinyl copolymers with controlled molecular weights and dispersities.^21,22^ (B) *Grafting-through* approach using light-mediated ROP of LA-functionalized poly(methyl methacrylate) macromonomers to synthesize degradable BB with disulfide-containing backbones.^20^ (C) In this work, degradable BB polymers were synthesized via ATRP *grafting-from* macroinitiators prepared through RAFT copolymerization of LA and BiBOEA inimer, followed by postpolymerization modification with HOBiB.

Inspired by this work, we developed a new synthetic strategy to prepare degradable BBs from LA with controlled molecular weights and low *Đ*, along with tunable chemical structure of side chains affording water solubility for potential use in biomedical applications (Figure 1C). Our approach begins with the RAFT copolymerization of LA and an acrylate-based initiator-monomer (inimer) 2-(2-(bromoisobutyryl)oxy)ethyl acrylate (BiBOEA), resulting in macroinitiator prepolymers (P(LA-*co*-BiBOEA)).^34^ They contain pendent α-bromoisobutyrate (BiB) initiating moieties for subsequent atom transfer radical polymerization (ATRP) *grafting-from* to obtain well-defined BB structures.^35,36^ We first investigated the kinetics and control of the RAFT copolymerization of LA and BiBOEA at a target DP of 200 and 25 mol % LA feed (Figure 2A). The polymerization was carried out in THF at 70 °C using *S*-dodecyl-*S*′-(α,α′-dimethyl-α′′-acetic acid)trithiocarbonate (DDMAT) as the chain transfer agent and azobis(isobutyronitrile) (AIBN) as the radical initiator.

**Figure 2:**
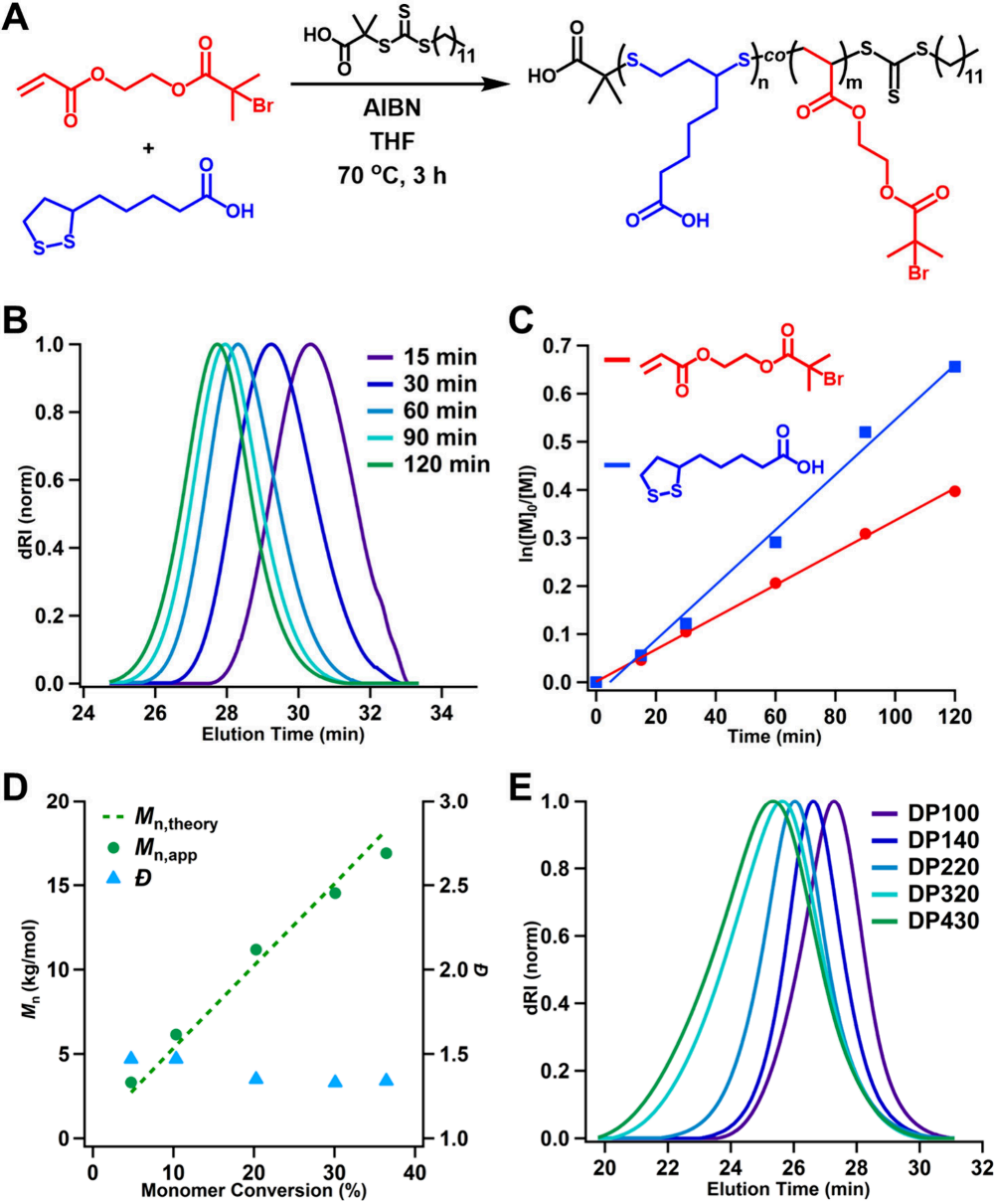
(A) Reaction scheme for the RAFT copolymerization of LA and BiBOEA. (B) Evolution of SEC traces over the course of the RAFT copolymerization of LA and BiBOEA showing time points taken between 0 and 120 min. (C) Pseudo-first order kinetic plot for conversions of both LA and BiBOEA during the RAFT copolymerization. (D) Plot of *M*_n,SEC_ versus total monomer conversion showing good agreement with *M*_n,theory_ while maintaining relatively controlled *Đ*. (E) SEC traces of P(LA-*co*-BiBOEA) for varying target DPs of 200–1000 and obtained DPs of 100–430.

Size exclusion chromatography with multiangle light scattering (SEC-MALS) analysis showed clean shifts of the SEC traces to lower elution times as the polymer chains grew uniformly during RAFT copolymerization (Figure 2B). The linear pseudo-first-order kinetic plot indicated constant radical concentration throughout the copolymerization and a faster incorporation rate of LA relative to BiBOEA consistent with the findings reported by Hawker et al. (Figure 2C). Moreover, apparent number-average molecular weight (*M*_n,app_) as determined by SEC showed a linear increase with conversion, aligning relatively well with the theoretical number-average molecular weight for quantitative initiation and monomer conversion, determined by ^1^H NMR spectroscopy (*M*_n,theory_) (Figure 2D). These results collectively demonstrated that RAFT copolymerization of LA and BiBOEA proceeded in a relatively controlled manner. The copolymerization was investigated for different target DPs (200, 300, 500, 700, 1000), and at 20 or 25 mol % feed of LA to ensure the incorporation of ample disulfide units in the backbones for degradation of the resulting BBs (Figure 2E). For all target DPs, after 3 h, BiBOEA conversion reached moderate values of 38–48% as measured by ^1^H NMR spectroscopy, while LA conversion reached >55%. LA incorporation in the purified polymers ranged from 29 to 35% (Table 1). For reaction times longer than 4 h, both comonomers reached slightly higher conversion, however an increase in *Đ* was observed. While dispersity remained relatively controlled (*Đ* = 1.36–1.51) for lower target backbone lengths (DP ≤ 500), it broadened for higher target DPs of 700 and 1000 (*Đ* = 1.94, 2.25), possibly due to some transfer to the polymer backbone. Nonetheless, SEC of the purified P(LA-*co*-BiBOEA) copolymers with varying DPs (100, 140, 220, 320, 430) revealed monomodal distributions for all copolymerizations, demonstrating predictable molecular weights and enabling the construction of backbones with higher *DP*_bb_ compared to previous reports.^17−20^

**Table 1:** Characterization of P(LA-*co*-BiBOEA) Polymers

entry	time (h)	LA (equiv)	BiBOEA (equiv)	LA conv.^a^ (%)	BiBOEA conv.^a^ (%)	LA incorp.^a^ (%)	*M*_n,theory_^b^ (kg/mol)	*M*_n,app_^c^ (kg/mol)	*Đ*^c^
1	4	50	150	66	48	33	26.2	22.8	1.41
2	3	75	225	59	44	29	34.3	30.7	1.36
3	3	100	400	55	41	30	55.2	40.5	1.51
4	3	175	525	59	41	34	78.6	53.6	1.94
5	3	250	750	55	38	35	104	59.9	2.25

a Monomer conversion and LA incorporation were determined by ^1^H NMR spectroscopy.

b Theoretical molar masses (*M*_n,theory_) were determined from the monomer conversion from ^1^H NMR spectroscopy.

c Relative number-average molar masses (*M*_n,app_) and dispersity (*Đ*) were determined by SEC analysis (THF as eluent) calibrated to poly(methyl methacrylate) standards.

To increase the density of ATRP initiator pendant groups on the macroinitiators and, by extension the side chain density of the BBs, we sought to install additional BiB units on the carboxylic acid pendant groups of LA (Figure 3A). Initially, a RAFT copolymerization of BiBOEA was attempted with an LA inimer containing a BiB moiety (Figure S1). This copolymerization yielded inconsistent results, proceeding to low conversions and multimodal molecular weight distributions (Figure S1). Therefore, the pendant carboxylic acids of the prepolymers were modified postpolymerization with 2-(hydroxyethyl) 2-bromoisobutyrate (HOBiB). From ^1^H NMR spectroscopy, the modification reached quantitative conversion (Figure 3B), and an increase in molecular weight was observed from SEC consistent with the addition of the BiB units (Figure 3C). All the P(LA-*co*-BiBOEA) polymers were subjected to this postpolymerization modification (Figures 3D, S2) and the resulting P(LABiB-*co*-BiBOEA) macroinitiators were used in ATRP *grafting-from* to synthesize degradable BB polymers.

**Figure 3:**
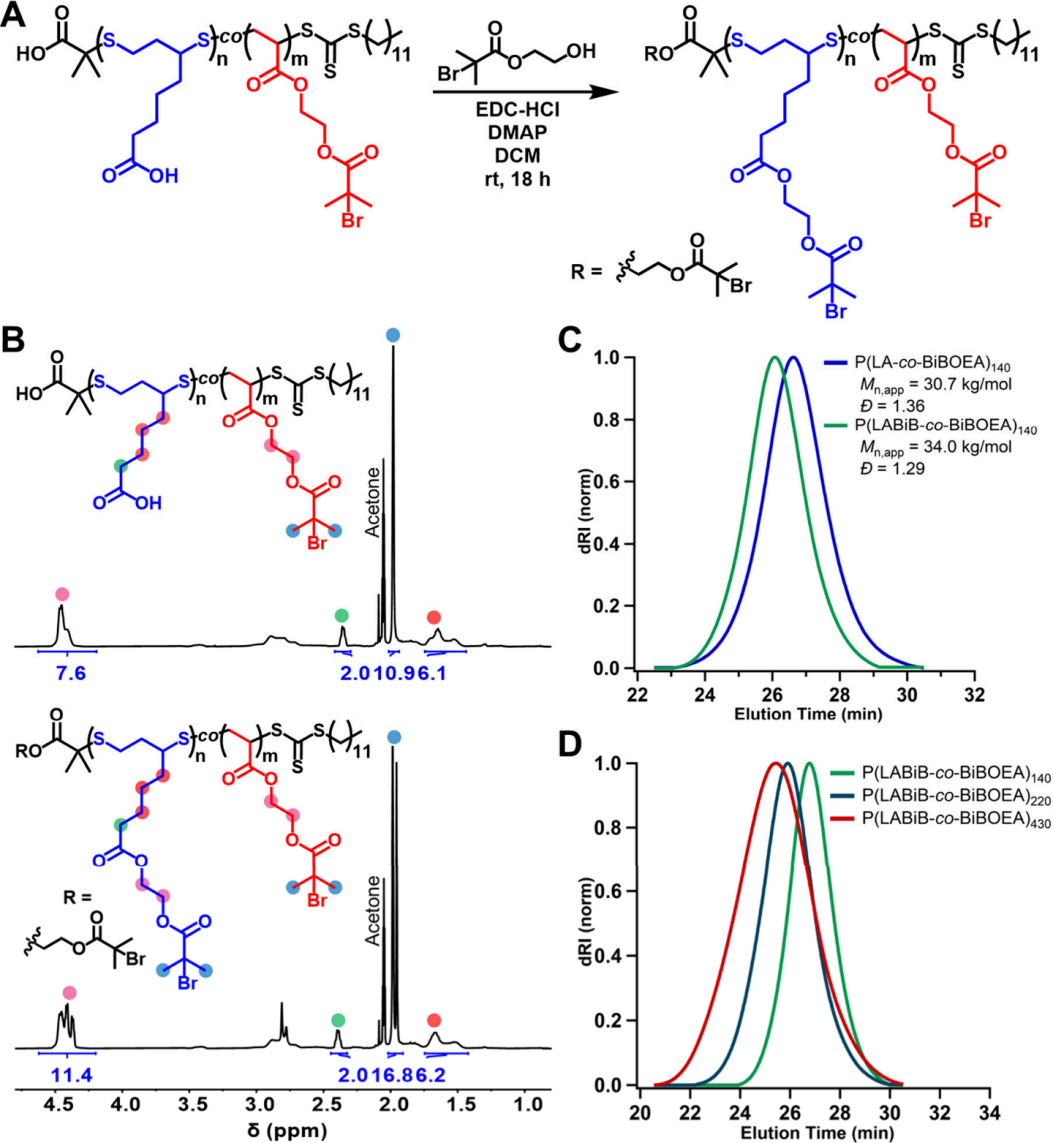
(A) Reaction scheme for the postpolymerization modification of P(LA-*co*-BiBOEA) prepolymers with HOBiB to obtain P(LABiB-*co*-BiBOEA) macroinitiators. (B) ^1^H NMR spectra of P(LA-*co*-BiBOEA) (top) and P(LABiB-*co*-BiBOEA) (bottom) recorded in acetone-*d*_6_. (C) Overlaid SEC traces of P(LA-*co*-BiBOEA)_140_ and P(LABiB-*co*-BiBOEA)_140_, showing an increase in molecular weight following postpolymerization modification. (D) SEC traces corresponding to P(LABiB-*co*-BiBOEA) macroinitiators of varying DP (140, 220, 430).

P(LABiB-*co*-BiBOEA)_100–430_ were used for ATRP *grafting-from* to obtain brushes with varying backbone chain lengths (*DP*_bb_). BA and tri(ethylene glycol) methyl ether acrylate (TEGA) were chosen as monomers (Figure 4A), with the latter serving to install hydrophilic side chains to provide water solubility. The *grafting-from* conditions were adapted from previously established normal ATRP conditions,^37,38^ with CuBr/CuBr_2_ catalyst and *N*,*N*,*N*′,*N*″,*N*″-pentamethyldiethylenetriamine (PMDTA) ligand in anisole at 60 °C. Using BA as the monomer, at molar ratios [BA]/[PMDTA]/[CuBr]/[CuBr_2_] = 400/0.6/0.53/0.03, the ATRP polymerizations reached moderate conversions of 12–27% following 23–24 h reaction times (Table 2), after which the polymerizations were stopped to maintain controlled dispersity and limit side chain length (*DP*_sc_). The resulting PBA brushes with *DP*_bb_ = 140, 220, and 430 had respective molar masses of 650, 1390, and 2290 kg/mol, as measured by SEC-MALS, and controlled dispersities comparable to the corresponding macroinitiators (*Đ* = 1.30, 1.42, 1.49) (Figure S3). The ATRP *grafting-from* was carried out using TEGA as the monomer with [TEGA]/[PMDTA]/[CuBr]/[CuBr_2_] = 400/0.6/0.53/0.03, in anisole and at 60 °C, obtaining PTEGA BB. These polymerizations proceeded much more rapidly than with BA, achieving conversions of 10–12% after 3–4 h (Table 2). SEC-MALS analysis of PTEGA BB with varying backbone lengths of *DP*_bb_ = 140, 220, and 430 revealed molar masses of 921, 1940, and 2700 kg/mol and *Đ* = 1.36, 1.37, 1.53, respectively (Figure 4C). The PTEGA BBs were water-soluble at concentrations up to 0.2% w/w, implying good biocompatibility that will be further enhanced by the degradability of the BB.

**Figure 4:**
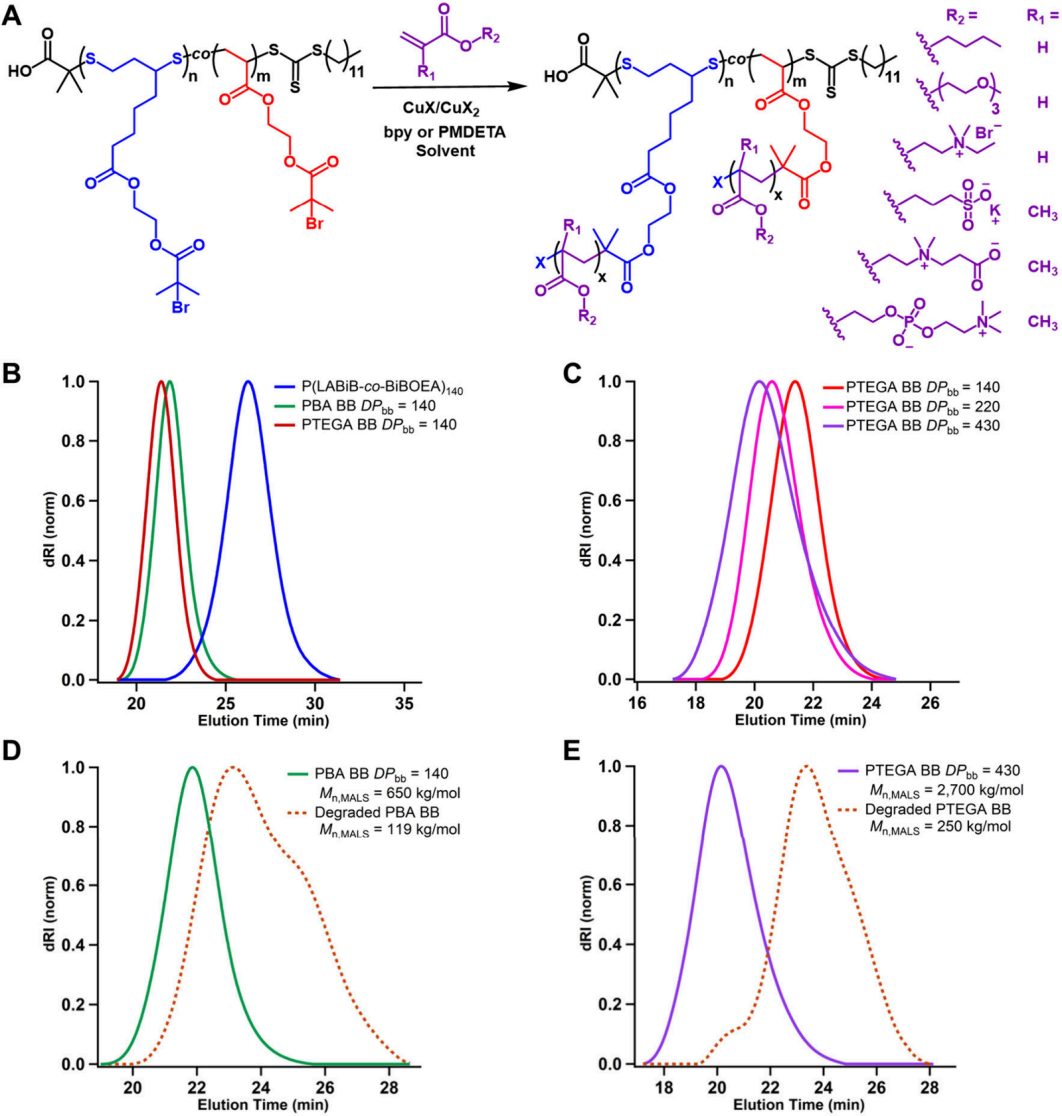
(A) Reaction scheme of ATRP *grafting-from* BB synthesis using various (meth-)acrylate monomers. (B) SEC trace of P(LABiB-*co*-BiBOEA)_140_ macroinitiator overlaid with corresponding traces of PBA and PTEGA BB after ATRP *grafting-from*. (C) PTEGA BB polymers of varying backbone lengths (*DP*_bb_). (D) SEC traces showing degradation of PBA BB using 0.5 M dithiothreitol (DTT) in DMF at 22 °C, after 24 h. (E) SEC traces showing degradation of PTEGA BB using 0.1 M tris(2-carboxyethyl) phosphine (TCEP) in water at 22 °C, after 24 h.

**Table 2:** Characterization of Degradable BB Polymers Obtained using ATRP *Grafting-From* P(LABiB-*co*-BiBOEA)

entry	MI DP	time (h)	monomer	conv.^a^ (%)	*M*_n,MALS_^b^ (kg/mol)	*Đ*^b^
1	140	23	BA	12	650	1.30
2	220	22	BA	27	1390	1.42
3	430	24	BA	17	2290	1.49
4	140	3.0	TEGA	10	921	1.36
5	220	4.5	TEGA	12	1940	1.37
6	430	3.0	TEGA	12	2700	1.53
7	220	4.0	DMEAA	8.0	4780	1.28
8	220	3.0	MPC	38	4070	1.35
9	320	1.0	CBMA	50	3700	1.16
10	100	1.0	SPMA	24	537	1.31

a Monomer conversion determined by ^1^H NMR spectroscopy.

b Absolute number-average molar mass (*M*_n,MALS_) and dispersity (*Đ)* were determined by SEC equipped with a multiangle light-scattering detector assuming 100% mass recovery, using DMF or aqueous buffer as eluent.

To expand the monomer scope of *grafting-from* ATRP and synthesize degradable brushes with charged and zwitterionic side chains bearing unique chemical and physical properties, several acrylate and methacrylate monomers were selected for *grafting-from* P(LABiB-*co*-BiBOEA) macroinitiators (Table 2 and Figure 4A). The quaternary ammonium-containing cationic *N*,*N*-dimethyl-*N*-ethylethylammonium acrylate (DMEAA) was used in ATRP *grafting-from* P(LABiB-*co*-BiBOEA)_220_, at ratios of [DMEAA]/[PMDTA]/[CuBr]/[CuBr_2_] = 400/0.6/0.5/0.03 and in DMSO as solvent. Within 2 h, the polymerization reached 8% conversion (Table 2), and the resulting BB polymer had a high molar mass (*M*_n,MALS_ = 4,780 kg/mol) with low dispersity (*Đ* = 1.28) (Figure S4). Furthermore, zwitterionic methacrylate monomers 2-methacryloyloxyethyl phosphorylcholine (MPC) and 3-[[2-(methacryloyloxy)ethyl]dimethylammonio] propionate (CBMA) were used in *grafting-from*, employing a previously reported CuCl/CuCl_2_ catalyst system with 2,2′-bipyridine (bpy) as ligand to ensure a controlled polymerization and prevent cross-link formation between BB structures.^39,40^ Zwitterionic monomers are of particular interest for biomedical applications due to their excellent antifouling properties, which minimize nonspecific protein adsorption and biofilm formation, making them ideal for applications such as drug delivery, biosensors, and implantable devices.^41,42^ Additionally, MPC-containing BB polymers have been reported to exhibit exceptional lubrication properties, mimicking the function of natural lubricants in articular cartilage, which reduces friction and wear under physiological conditions.^39,43^ However, these zwitterionic BB polymers, including MPC-based systems, have not yet been made degradable, limiting their potential for applications requiring controlled breakdown. When carried out at 50 °C in methanol/DMSO solvent mixture at [methacrylate]/[bpy]/[CuCl]/[CuCl_2_] ratios = 125/3.40/1.50/0.20, the ATRP with MPC reached 38% conversion. The molar mass of the PMPC BB was measured at 4070 kg/mol, and dispersity remained controlled at *Đ* = 1.35 (Figure S4). Similarly, the ATRP with CBMA reached 50% conversion in just 1 h at [methacrylate]/[bpy]/[CuCl]/[CuCl_2_] ratios = 150/3.40/1.50/0.20 using methanol/DMSO/acetonitrile solvent mixture at 40 °C, with the molar mass of the PCBMA BB being measured at 3,700 kg/mol, and its dispersity at *Đ* = 1.16. Finally, the anionic monomer 3-sulfopropyl methacrylate (SPMA) was used in ATRP *grafting-from* P(LABiB-*co*-BiBOEA)_100_ at ratios of [methacrylate]/[bpy]/[CuCl]/[CuCl_2_] = 150/3.40/1.50/0.20 in DMSO solvent and reached 24% conversion in 1h. The molar mass of the PSMPA BB was measured at 537 kg/mol, and its dispersity at *Đ* = 1.31.

The degradation of the BBs was investigated using mild thiol reducing agents dithiothreitol (DTT) and TCEP. Using 0.5 M DTT in DMF at 22 °C, the PBA brush with *DP*_bb_ = 140 (*M*_n,MALS_ = 650 kg/mol, *Đ* = 1.30) degraded to 18% of its original molar mass (*M*_n,MALS_ = 119 kg/mol, *Đ* = 1.53) after 24 h (Figure 4D). Aqueous TCEP (0.1 M) readily degraded PTEGA brush with *DP*_bb_ = 430 (*M*_n,MALS_ = 2700 kg/mol, *Đ* = 1.53) at 22 °C to just 9% of its molar mass (*M*_n,MALS_ = 250 kg/mol, *Đ* = 1.87) after 24 h (Figure 4E). The ability of both reducing agents to degrade the BB confirmed the presence of disulfide bonds in the backbones, with the more complete degradation by TCEP reflecting its greater power as a reducing agent (*E*^*o*^ = −1.62 V) compared to DTT (*E*^o^ = −0.33 V) and the lack of disulfide bond reformation after reduction.^44,45^ These favorable degradation profiles using mild reducing agents further support the future application of the BB polymers in applications requiring biocompatibility and degradation after use.

In summary, we have developed a synthetic strategy for degradable BB polymers with backbone disulfide bonds. α-Lipoic acid (LA) was copolymerized through RAFT with ATRP inimer 2-(2-(bromoisobutyryl)oxy)ethyl acrylate (BiBOEA) to yield prepolymers that could be further modified with 2-(hydroxyethyl) 2-bromoisobutyrate (HOBiB) to obtain ATRP macroinitiators with high initiator side chain density. ATRP *grafting-from* was employed to synthesize BB polymers with varying molecular weight using *n*-butyl acrylate (BA), as well as hydrophilic tri(ethylene glycol) methyl ether acrylate (TEGA) for water solubility. In addition, a cationic acrylate and zwitterionic methacrylates were used as monomers in ATRP *grafting-from*, entailing a broad monomer scope for obtaining water-soluble degradable BBs with charged side chains for biomedical applications. Finally, the BB polymers were degraded under mild thiol-reducing conditions using dithiothreitol (DTT) and 2-tris(2-carboxyethyl) phosphine, confirming the presence of degradable disulfide units in the backbone that enhance the biocompatibility of the BBs.

## Supporting Information Available

Chemicals, instrumentation, synthetic procedures, size-exclusion chromatograms, and ^1^H NMR spectra (PDF)
