# Supplementary material for: Versatile and Controlled Synthesis of Degradable, Water-Soluble Bottlebrush Polymers with Poly(disulfide) Backbones Derived from α-Lipoic Acid
Source: ACS Macro Lett. 2025 Feb 3;14(2):207–13. doi: 10.1021/acsmacrolett.4c00839 (PMC11841036; doi:10.1021/acsmacrolett.4c00839)
Supplement: Supplementary file 1 — mz4c00839_si_001.pdf [file mz4c00839_si_001.pdf]

Supporting Information for

Versatile and Controlled Synthesis of Degradable, Water-Soluble Bottlebrush Polymers with Poly(disulfide) Backbones Derived from *α*-Lipoic Acid

Ivan O. Levkovsky, Lucca Trachsel, Hironobu Murata, Krzysztof Matyjaszewski*

Department of Chemistry, Carnegie Mellon University, Pittsburgh, Pennsylvania 15213, USA

E-mail: km3b@andrew.cmu.edu

Supporting Information:

INSTRUMENTATION 2
SYNTHESIS 2
SUPPLEMENTARY FIGURES 6
REFERENCES 16

Instrumentation

Nuclear Magnetic Resonance (NMR) Spectroscopy. ^{1}H NMR spectra were collected using a Bruker Advance 500 MHz NMR spectrometer. Deuterated acetone (acetone-d_6), deuterated chloroform ($CDCl_3$), and deuterium oxide (D_2O) were used as solvents, and the residual solvent signal served as a reference.

Size-Exclusion Chromatography (SEC). The apparent number-average molecular weights ($M_{n,app}$) and dispersity ($Đ$) of P(LA-*co*-BiBOEA) and P(LABiB-*co*-BiBOEA) polymers were measured relative to poly(methyl methacrylate) (PMMA) standards using an Agilent 1260 Infinity II isocratic pump, a column set containing 3 PSS analytical columns (Styrogel 10^2, 10^3, 10^4, 10^5 Å pore sizes), and Agilent 1260 Infinity II refractive index (RI) detector with THF as eluent at 35 °C and a flow rate of 1 mL/min.

SEC-MALS (multi-angle light scattering) analysis for PBA and PTEGA bottlebrush (BB) polymers were preformed using an Agilent 1260 Infinity II isocratic pump, 3 PSS columns (Styrogel 10^5, 10^3, 10^2 Å), an Agilent 1260 Infinity II RI detector, and a PSS SLD2020 MALS detector with DMF + 50 mM LiBr as an eluent at 50 °C and a flow rate of 1 mL/min. Absolute number-average molecular weights ($M_{n,abs}$) and dispersities were determined using refractive index increment (dn/dc) values acquired via off-line analysis and 100% mass recovery methods.

SEC-MALS characterizations for PDMEAA, PMPC, and PCBMA BB polymers were performed using Agilent SEC system (1260 Infinity II) equipped with UV detector, DAWN HELEOS-II (Wyatt) MALS detector and Optilab T-rEX (Wyatt Technology) RI detector. Measurements of PMPC and PCBMA bottlebrush polymers were performed using a SUPREMA Lux 3000 Å, 8 × 300 mm, 10 µm and guard column (PSS) at a flow rate of 0.5 mL/min with Dulbecco's Phosphate Buffered Saline and 0.02 wt% sodium azide as eluent. Measurement of PDMEAA bottlebrush polymer was performed using a NOVEMA Max Lux 3000 Å, 8 × 300 mm, 10 µm and guard column (PSS) at a flow rate of 0.5 mL/min with the mixture of 10 mM sodium phosphate (pH 4.5) and 100 mM NaCl as eluent.

Synthesis

Materials. All reagents and solvents were purchased at the highest commercial grade and used as received unless otherwise noted. Azobisisobutyronitrile (AIBN, Millipore Sigma, 98%) was recrystallized two times from methanol and dried under high vacuum prior to use. *n*-Butyl acrylate (BA, Millipore Sigma, ≥99%) and tri(ethylene glycol) methyl ether acrylate (TEGA, TCI, >90.0%) were passed through a basic alumina column to remove inhibitors and acidic impurities, and were stored at -20 °C. 2-Methacryloxylethyl phorsphorylcholine (MPC, Millipore Sigma, 97%) was recrystallized from acetonitrile and dried under high vacuum prior to use. Copper(I) bromide (CuBr, Millipore Sigma, 98%) and copper(I) chloride (CuCl, Millipore Sigma, 97%) were purified by stirring in glacial acetic acid overnight, washing with copious amounts of absolute ethanol and then diethyl ether, and finally dried under high vacuum overnight. Tetrahydrofuran (THF, Fisher, ACS grade) and 1,4-dioxane (Fisher, ACS grade) were passed through a column of basic alumina to remove inhibitors and adventitious peroxides prior to polymerization. 2-2-(hydroxyethyl)bromoisobutyrate (HOBiB),[1] (S)-1-doceyl-(S)-(α,α′-dimethyl-α″-acidic acid) trithiocarbonate (DDMAT),[2] and *N*-ethyl-*N,N*-dimethylethylammonium acrylate (DMEAA)[3] were synthesized as reported previously. Deuterated chloroform ($CDCl_3$, 99.9%), deuterated acetone (acetone-d_6, 99.9%), %), and deuterium oxide (D_2O, 99.9%) were obtained from Cambridge Isotope Laboratories.

2-(2-(bromoisobutyryl)oxy)ethyl acrylate (BiBOEA). In a 1 L round bottom flask, 2-hydroxyethyl acrylate (15.65 g, 139 mmol, 1 equiv) was dissolved in dichloromethane (DCM, 300 mL) and triethylamine (14.03 g, 139 mmol, 1 equiv) was added. Under argon atmosphere, α-bromoisobutyryl bromide (31.88 g, 139 mmol, 1 equiv) in DCM (60 mL) was added dropwise over 3 h at 0 °C and stirred overnight at room

temperature. The reaction mixture was then added to a separatory funnel and washed with 1 M HCl (3 x 250 mL), water (1 x 200 mL), saturated sodium bicarbonate (3 x 250 mL), and brine (200 mL), and then was finally dried using anhydrous magnesium sulfate. Following filtration of the solution and removal of the DCM, the crude product was obtained as a slightly yellow oil, which was further purified via vacuum distillation to yield a colorless oil (26.46 g, 100 mmol, 72.0% yield). ^{1}H NMR: (500 MHz, $CDCl_3$,) δ 6.46 (dd, J = 17.3, 1.4 Hz, 1H), 6.16 (dd, J = 17.3, 10.4 Hz, 1H), 5.89 (dd, J = 10.4, 1.4 Hz, 1H), 4.44 (s, 4H), 1.95 (s, 6H).

2-(2-(Bromoisobutyryl)oxy)ethyl α-lipoate (LABiB). In a 250 mL round bottom flask, α-lipoic acid (1.00g, 1 equiv, 4.85 mmol) was dissolved in 100 mL DCM. 4-dimethylaminopyridine (DMAP, 296 mg, 0.5 equiv, 2.42 mmol) and 1-ethyl-3-(3-dimethylaminopropyl)carbodiimide hydrochloride (EDC-HCl, 1.39 g, 1.5 equiv, 7.27 mmol) was added, and the solution was strongly stirred for 15 min until all solids disappeared. HOBiB (1.23 g, 5.82 mmol, 1.2 equiv) was added dropwise to the mixture, which was then stirred overnight at room temperature. The reaction mixture was then added to a separatory funnel and washed with 1 M HCl (3 x 250 mL), water (1 x 200 mL), saturated sodium bicarbonate (3 x 250 mL), and brine (200 mL), and then was finally dried using anhydrous magnesium sulfate. Following filtration of the solution and removal of the DCM, the crude product was obtained as an opaque, bright yellow viscous oil, which was purified using silica column chromatography eluting with ethyl acetate: hexanes (1:4 v/v) to yield a transparent, bright yellow viscous oil (1.61 g, 83% yield). The product was prone to autopolymerization during storage at room temperature and at –20 °C after 2–3 days. ^{1}H NMR: (500 MHz, $CDCl_3$) δ 4.47 – 4.21 (m, 4H), 3.58 (dq, J = 8.5, 6.4 Hz, 1H), 3.26 – 3.01 (m, 2H), 2.48 (dtd, J = 13.0, 6.6, 5.3 Hz, 1H), 2.37 (t, J = 7.4 Hz, 2H), 1.95 (s, 7H), 1.79 – 1.63 (m, 4H), 1.58 – 1.3°°9 (m, 2H).

Attempted copolymerization of LABiB and BiBOEA. In a 4 mL glass vial, LABiB (180 mg, 450 μmol, 50 equiv) was combined with BiBOEA (358 mg, 1.35 mmol, 150 equiv). DDMAT was then added (3 mg, 9 μmol, 1 equiv), followed by 20 μL of a 11 mg/mL stock solution of AIBN in 1,4-dioxane (222 μg, 1.4 μmol, 0.15 equiv). The vial was fitted with a rubber septum, and the polymerization mixture was sparged with argon for 20 min. The vial was placed in an oil bath at 70 °C, with monomer conversion being monitored using ^{1}H NMR spectroscopy. Monomer conversion proceeded to reach 25% for BiBOEA, and 38% for LA after 23 h. THF SEC analysis revealed multimodal molecular weight distribution for the polymer and relatively broad dispersity ($M_{n,theory}$ = 18.0 kg/mol, $M_{n,app}$ = 16.7 kg/mol, $Đ$ = 1.83).

General synthesis of P(LA-co-BiBOEA). In a 4 mL glass vial, LA (220 mg, 1.07 mmol, 75 equiv) was dissolved in 330 μL THF. BiBOEA (848 mg, 3.20 mmol, 225 equiv) and DDMAT (5 mg, 14 μmol, 1 equiv). A small crystal of AIBN was added, and the vial was fitted with a rubber septum. The polymerization mixture was sparged with argon for 20 min. The vial was placed in an oil bath at 70 °C, with monomer conversion being monitored using ^{1}H NMR spectroscopy. Once conversions of BiBOEA and LA reached >40% and >55%, respectively, the reaction rate drastically slowed, and the polymerization was stopped by exposure to air and dilution with acetone. The polymer was purified via precipitation from acetone into an ice-cold 1:1 mixture of diethyl ether/hexanes three times and dried on high vacuum resulting in a light yellow solid (310 mg, 64%) ($M_{n,theory}$ = 34.3 kg/mol, $M_{n,app}$ = 30.7 kg/mol, $Đ$ = 1.36).

P(LA-co-BiBOEA) polymerization kinetics. In a 4 mL glass vial LA (448 mg, 2.17 mmol, 50 equiv) was dissolved in 680 μL, and BiBOEA (1.73 g, 6.51 mmol, 150 equiv) was added (total [M] = 4.1 M). DDMAT (15.8 mg, 43 μmol, 1 equiv) and AIBN (1 mg, 6.5 μmol, 0.15 equiv) were added to the reaction mixture, and the vial was fitted with a rubber septum. After sparging with argon for 20 min, the vial was placed an oil bath at 70 °C, and the polymerization progress was monitored by taking 100 μL from the reaction mixuture at different timepoints, and using 50 μL for ^{1}H NMR spectroscopy in acetone-d_6 solvent and 50 μL for THF SEC.

General synthesis of P(LABiB-co-BiBOEA). In a 20 mL glass vial, 310 mg P(LA-*co*-BiBOEA) ($M_{n,app}$ = 30.7 kg/mol, $M_{n,SEC}$ = 30.7 kg/mol, $Đ$ = 1.36, 680 μmol carboxylic acids, 1.0 equiv) was dissolved in 5 mL DCM. DMAP (42 mg, 340 μmol, 0.5 equiv) was added, followed by slow addition of EDC-HCl (197 mg, 1.00 mmol, 1.5 equiv) with stirring. After all solids had dissolved, HOBiB (173 mg, 821 μmol, 1.2 equiv) was added, and the reaction was stirred overnight at room temperature. The polymer was purified via precipitation from DCM into ice-cold methanol three times, and then dried on high vacuum resulting in a light yellow solid ($M_{n,theory}$ = 41.2 kg/mol, $M_{n,app}$ = 34.0 kg/mol, $Đ$ = 1.29).

General synthesis of PBA bottlebrushes. In an oven-dried 10 mL Schlenk flask P(LABiB-*co*-BiBOEA) ($M_{n,app}$ = 34.0 kg/mol, $Đ$ = 1.29, 26 mg, 85 μmol bromo isobutyrate initiating groups, 1.0 equiv) was dissolved in 1 mL anisole. 13 μL of a 44 mg/mL stock solution of copper(II) bromide in DMSO (574 μg, 2.6 μmol, 0.03 equiv) was added, followed by 10.8 μL of PMDTA (9 mg, 52 μmol, 0.53 equiv) and 5.0 mL BA (4.45 g, 34.3 mmol, 400 equiv). The reaction mixture was deoxygenated using 3 freeze-pump-thaw cycles. During the final cycle, the flask was backfilled with argon, and CuBr (6 mg, 43 μmol, 0.5 equiv) was quickly added to the frozen reaction mixture. The flask was resealed, then degassed and backfilled with argon 10 times. The flask was placed in a 60 °C oil bath, with monomer conversion being monitored using ^{1}H NMR spectroscopy. After 24 h, the polymerization had reached the desired conversion and was stopped by dilution with DCM and exposure to air. After evaporation of 90% of the DCM volume, the bottlebrush polymer was purified by precipitation into ice-cold 3:7 water/methanol mixture 5 times to yield a white solid.

General synthesis of PTEGA bottlebrushes. In an oven-dried 25 mL Schlenk flask P(LABiB-*co*-BiBOEA) ($M_{n,app}$ = 34.0 kg/mol, $Đ$ = 1.29, 26 mg, 85 μmol bromo isobutyrate initiating groups, 1.0 equiv) was dissolved in 2 mL anisole. 13 μμL of a 44 mg/mL stock solution of copper(II) bromide in DMSO (574 μg, 2.6 μmol, 0.03 equiv) was added, followed by 10.8 μL of PMDTA (9 mg, 52 μmol, 0.53 equiv) and 7.1 mL TEGA (7.5 g, 34.3 mmol, 400 equiv). The reaction mixture was deoxygenated using 3 freeze-pump-thaw cycles. During the final cycle, the flask was backfilled with argon, and CuBr (6.0 mg, 43 μmol, 0.5 equiv) was quickly added to the frozen reaction mixture. The flask was recapped, evacuated, and backfilled with argon 10 times. The flask was placed in a 60 °C oil bath, with monomer conversion being monitored using ^{1}H NMR spectroscopy. After 4 h, the polymerization had reached the desired conversion and was stopped by dilution with DCM and exposure to air. After evaporation of 90% of the DCM volume, the bottlebrush polymer was purified by precipitation from DCM into ice-cold hexanes a white solid.

PDMEAA bottlebrush. In an oven-dried 10 mL Schlenk flask P(LABiB-*co*-BiBOEA) ($M_{n,app}$ = 47.9 kg/mol, $Đ$ = 1.42, 6 mg, 20 μmol bromo isobutyrate initiating groups, 1.0 equiv) was dissolved in 1 mL DMSO. In a separate vial, DMEAA (2.01 g, 8.01 mmol, 400 equiv) was dissolved in 4 mL DMSO, and then the solution was added to the Schlenk flask, along with 13 μL of a 10 mg/mL stock solution of copper(II) bromide in DMSO (132 μg, 0.60 μmol, 0.03 equiv) and 8 μL of a 250 mg/mL stock solution of PMDTA in DMSO (2.07 mg, 12 μmol, 0.53 equiv). The reaction mixture was deoxygenated using 3 freeze-pump-thaw cycles. During the final cycle, the flask was backfilled with argon, and CuBr (1.5 mg, 10 μmol, 0.5 equiv) was quickly added to the frozen reaction mixture. The flask was resealed, then degassed and backfilled with argon 10 times. The flask was placed in a 60 °C oil bath, with monomer conversion being monitored using ^{1}H NMR spectroscopy. After 4 h, the polymerization had reached the desired conversion and was stopped by dilution with deionized (DI) water and exposure to air. The bottlebrush polymer was purified by precipitation from DI water into ice-cold THF 5 times to yield a slightly yellow solid.

PMPC bottlebrush. In an oven-dried 10 mL Schlenk flask P(LABiB-*co*-BiBOEA) ($M_{n,app}$ = 47.9 kg/mol, $Đ$ = 1.42, 10 mg, 33 μmol bromo isobutyrate initiating groups, 1.0 equiv) was dissolved in 1 mL DMSO. In a separate vial, MPC (1.21 g, 4.09 mmol, 125 equiv) was dissolved in 6 mL of methanol/acetonitrile (1/1) and added to the Schleck flask. 23 μL of a 16 mg/mL stock solution of copper(II) chloride in DMSO was added

to the Schlenk flask (368 µg, 2.7 µmol, 0.2 equiv), along with of 2,2'-bipyridine (bpy, 17 mg, 110 µmol, 3.4 equiv). The reaction mixture was deoxygenated using 3 freeze-pump-thaw cycles. During the final cycle, the flask was backfilled with argon, and CuCl (5 mg, 50 µmol, 1.5 equiv) was quickly added to the frozen reaction mixture. The flask was resealed, then degassed and backfilled with argon 10 times. The flask was placed in a 60 °C oil bath, with monomer conversion being monitored using ^{1}H NMR spectroscopy. After 3 h, the polymerization had reached the desired conversion and was stopped by dilution with deionized (DI) water and exposure to air. The bottlebrush polymer was purified by dialysis against DI water using regenerated cellulose membrane with 50 kDa molecular weight cutoff. After lyophilization, the bottlebrush was obtained as a white solid.

PCBMA bottlebrush. In an oven-dried 25 mL Schlenk flask P(LABiB-*co*-BiBOEA) ($M_{n,app}$ = 56.0 kg/mol, $Đ$ = 1.88, 11 mg, 36 µmol bromo isobutyrate initiating groups, 1.0 equiv) was dissolved in 0.6 mL DMSO. In a separate vial, CBMA (1.24 g, 5.40 mmol, 150 equiv) was dissolved in 5 mL methanol and added to the Schlenk flask. 3 mL acetonitrile and 2.4 mL DMSO were then added to the Schleck flask to solubilize both the P(LABiB-*co*-BiBOEA) and CBMA. 59.4 µL of a 16 mg/mL stock solution of copper(II) chloride in DMSO was added to the Schlenk flask (950 µg, 7.2 µmol, 0.2 equiv), along with bpy (19.1 mg, 122 µmol, 3.40 equiv). The reaction mixture was deoxygenated using 3 freeze-pump-thaw cycles. During the final cycle, the flask was backfilled with argon, and CuCl (5.2 mg, 53.0 µmol, 1.5 equiv) was quickly added to the frozen reaction mixture. The flask was resealed, then degassed and backfilled with argon 10 times. The flask was placed in a 40 °C oil bath, with monomer conversion being monitored using ^{1}H NMR spectroscopy. After 1h, the polymerization had reached the desired conversion and was stopped by dilution with deionized (DI) water and exposure to air. The bottlebrush polymer was purified by dialysis against DI water in an RC membrane with 50 kDa molecular weight cutoff. After lyophilization, the bottlebrush was obtained as a white solid.

PSPMA bottlebrush. In an oven-dried 10 mL Schlenk flask P(LABiB-*co*-BiBOEA) ($M_{n,app}$ 28.0 kg/mol, $Đ$ = 1.41, 6.1 mg, 20 µmol bromo isobutyrate initiating groups, 1.0 equiv) was dissolved in 1 mL DMSO. In a separate vial, SPMA (737 mg, 2.99 mmol, 150 equiv) was dissolved in 4 mL DMSO and added to the Schlenk flask. 33.5 µL of a 16 mg/mL stock solution of copper(II) chloride in DMSO was added to the Schlenk flask (536 µg, 3.99 µmol, 0.2 equiv), along with bpy (11.2 mg 71.8 µmol, 3.4 equiv). The reaction mixture was deoxygenated using 3 freeze-pump-thaw cycles. During the final cycle, the flask was backfilled with argon, and CuCl (3.0 mg, 29.9 µmol, 1.5 equiv) quickly added to the frozen reaction mixture. The flask was placed in a 40 °C oil bath, with monomer conversion being monitored using ^{1}H NMR spectroscopy. After 1h, the polymerization had reached the desired conversion and was stopped by dilution with deionized (DI) water and exposure to air. The bottlebrush polymer was purified by dialysis against DI water in an RC membrane with 50 kDa molecular weight cutoff. After lyophilization, the bottlebrush was obtained as a white solid.

Degradation of PBA bottlebrush with DTT. In a 20 mL glass vial, PBA bottlebrush polymer ($M_{n,MALS}$ = 650 kg/mol, $Đ$ = 1.30, 60 mg) was dissolved in *N,N*-dimethylformamide (DMF, 6 mL). Dithiothreitol (DTT, 231 mg, 15.0 mmol) was added, and the mixture was stirred at room temperature. Change in molecular weight being monitored by taking 100 µL of the mixture and analyzing via SEC-MALS. The degradation was stopped after no more change in molecular weight was observed.

Degradation of PTEGA bottlebrush with TCEP. In a 20 mL glass vial, PTEGA bottlebrush polymer ($M_{n,MALS}$ = 2,700 kg/mol, $Đ$ = 1.53, 60 mg) was dissolved in 6 mL DI water. Tris(2-carboxyethyl) phosphine (TCEP, 172 mg, 922 µM) was added, and the mixture was stirred at room temperature. Change in molecular weight being monitored by taking 100 µL of the mixture and analyzing via SEC-MALS. The degradation was stopped after no more change in molecular weight was observed.

Supplementary Figures:

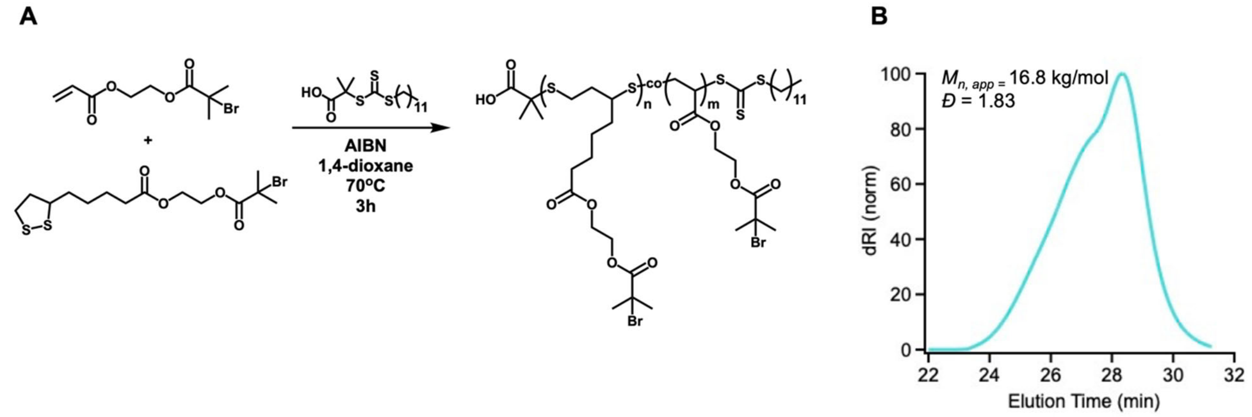

Figure S1. (A) Scheme for the attempted copolymerization of LABiB and BiBOEA. (B) SEC trace of P(LABiB-*co*-BiBOEA) after 23 h reaction time.

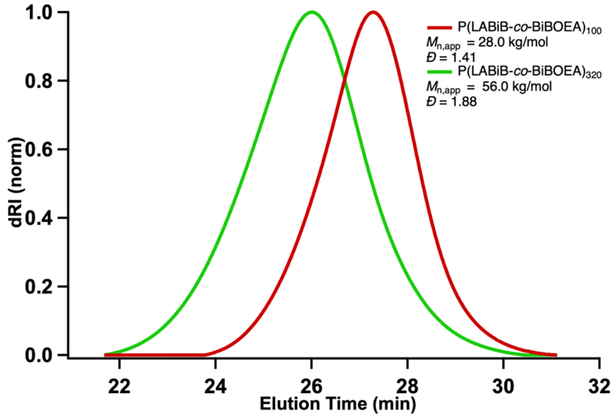

Figure S2. SEC traces corresponding to P(LABiB-*co*-BiBOEA)$_{100}$ and P(LABiB-*co*-BiBOEA)$_{320}$ macroinitiators.

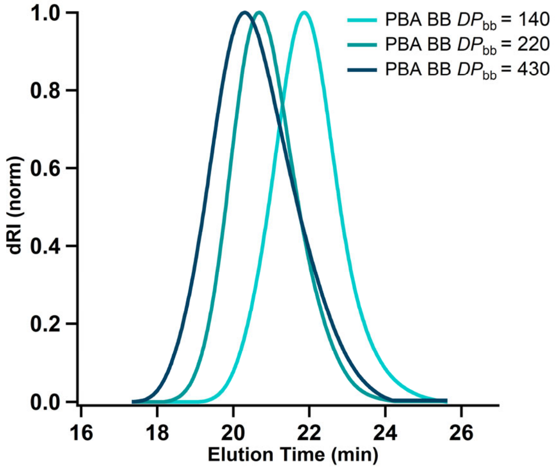

Figure S3. Overlaid SEC traces of PTEGA BB polymers of varying backbone lengths (DP_{bb}).

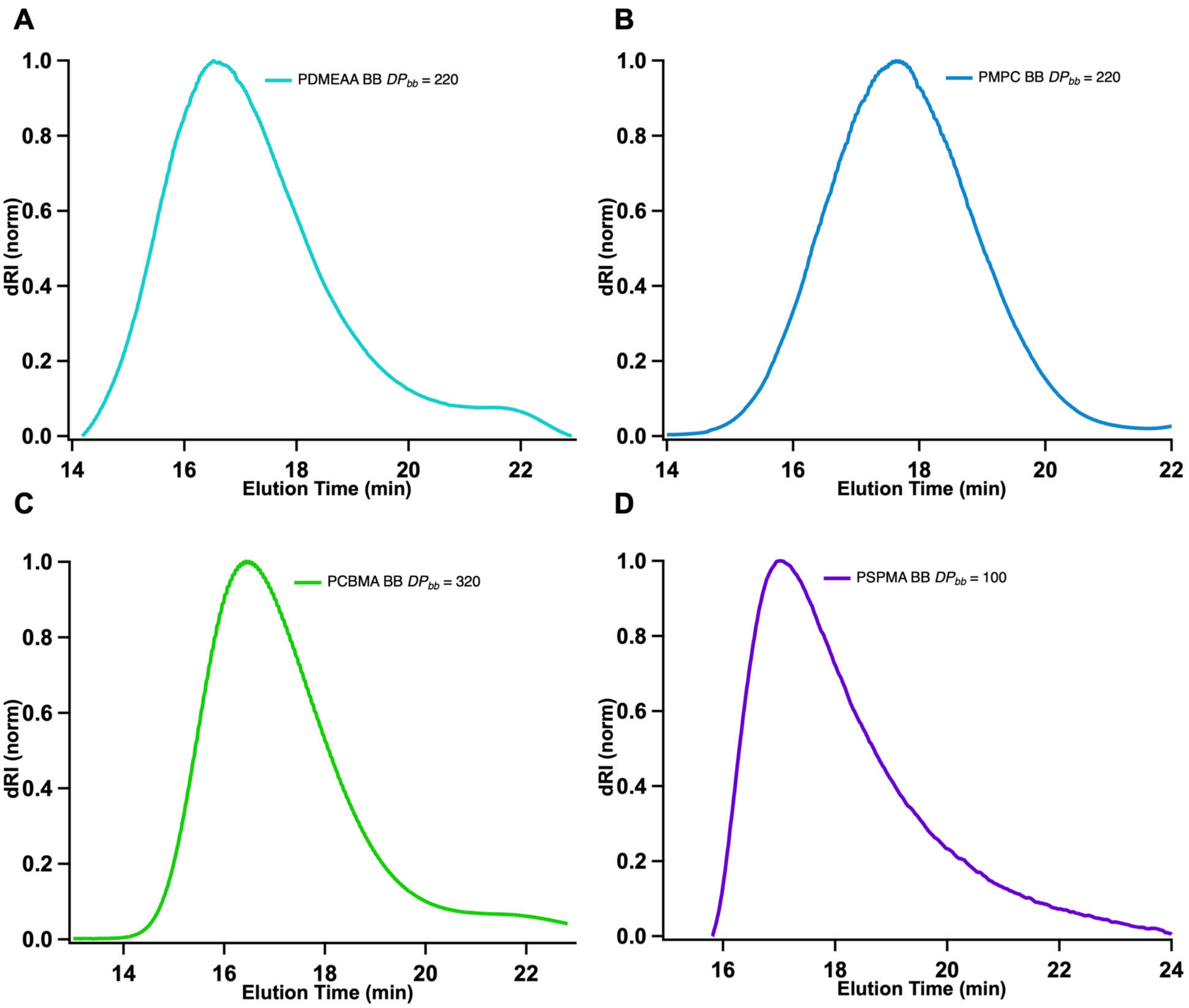

Figure S4: (A) SEC trace of poly(2-methacryloxylethyl phorsphorylcholine) (PMPC) BB. (B) SEC trace of poly(*N*-ethyl-*N,N*-dimethylethylammonium acrylate) (PDMEAA) BB. (C) SEC trace of poly(3-[[2-(methacryloyloxy) ethyl]dimethylammonio]propionate) (PCBMA) BB. (D) SEC trace of poly(3-sulfopropyl methacrylate) (PSPMA) BB.

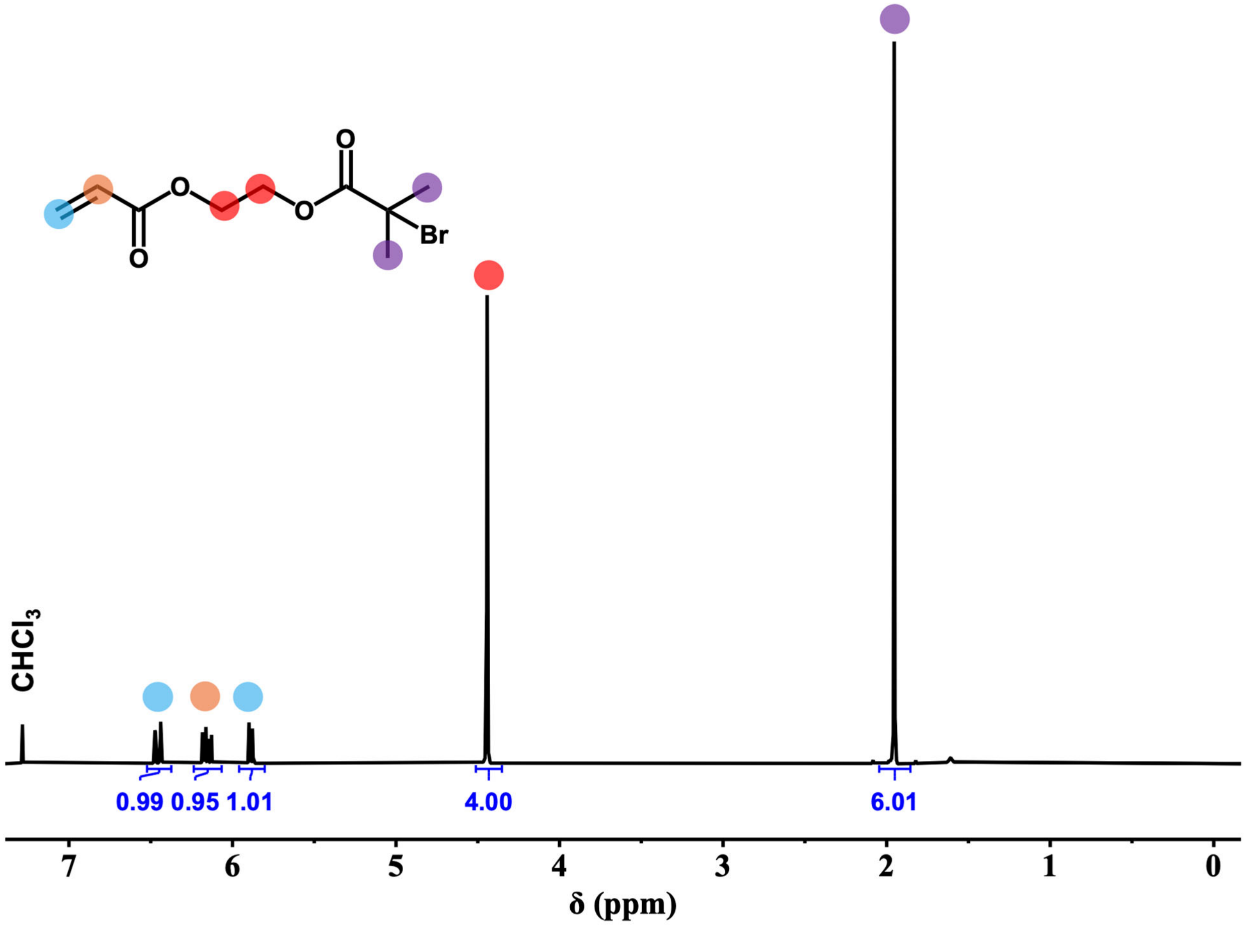

Figure S5. ^{1}H NMR spectrum (500 MHz) of 2-(2-(bromoisobutyryl)oxy)ethyl acrylate (BiBOEA) recorded in $CDCl_3$

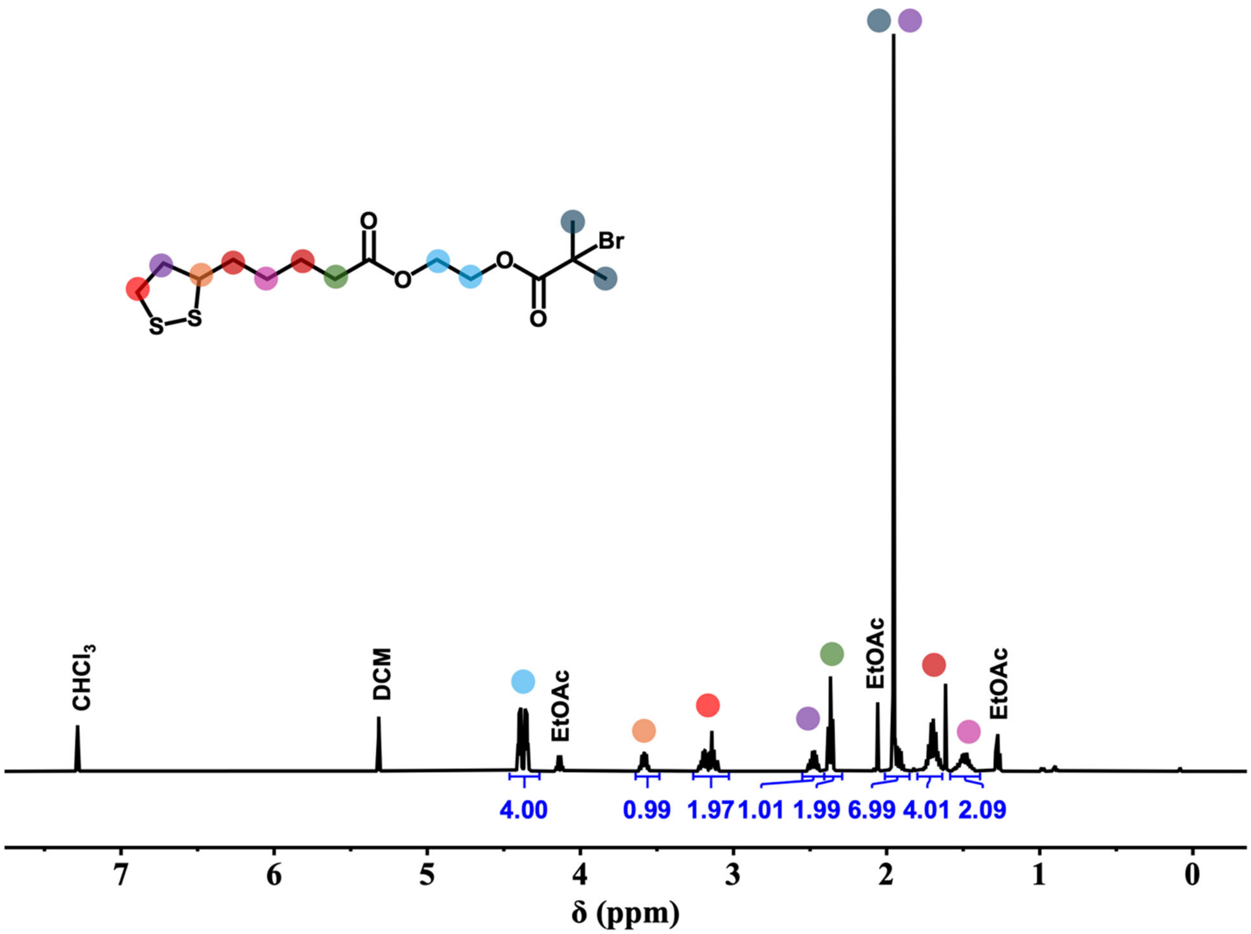

Figure S6. ^{1}H NMR spectrum (500 MHz) of 2-(2-(bromoisobutyryl)oxy)ethyl α-lipoate (LABiB) recorded in $CDCl_3$

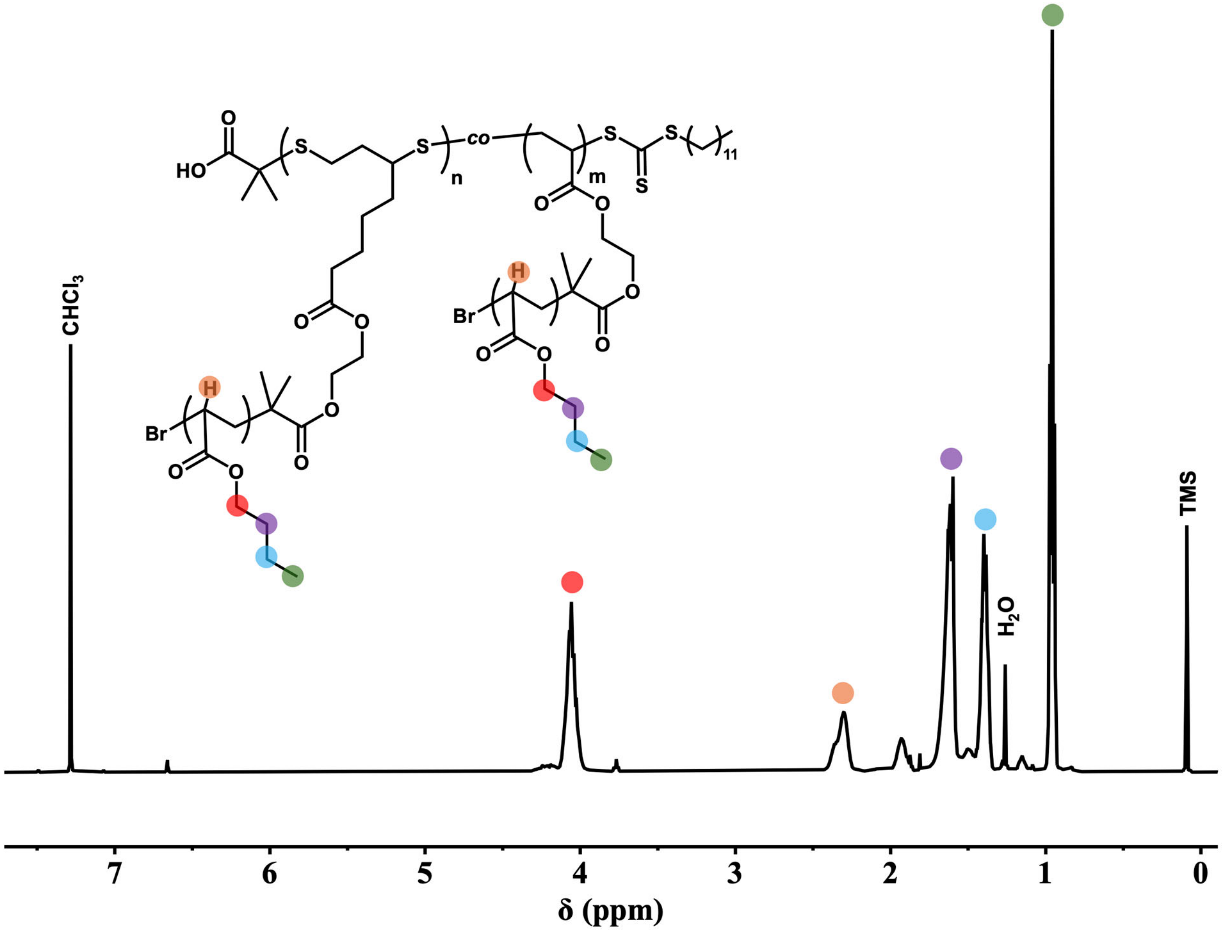

Figure S7. ^{1}H NMR spectrum (500 MHz) of poly(*n*-butyl acrylate) bottlebrush (PBA) BB recorded in $CDCl_3$.

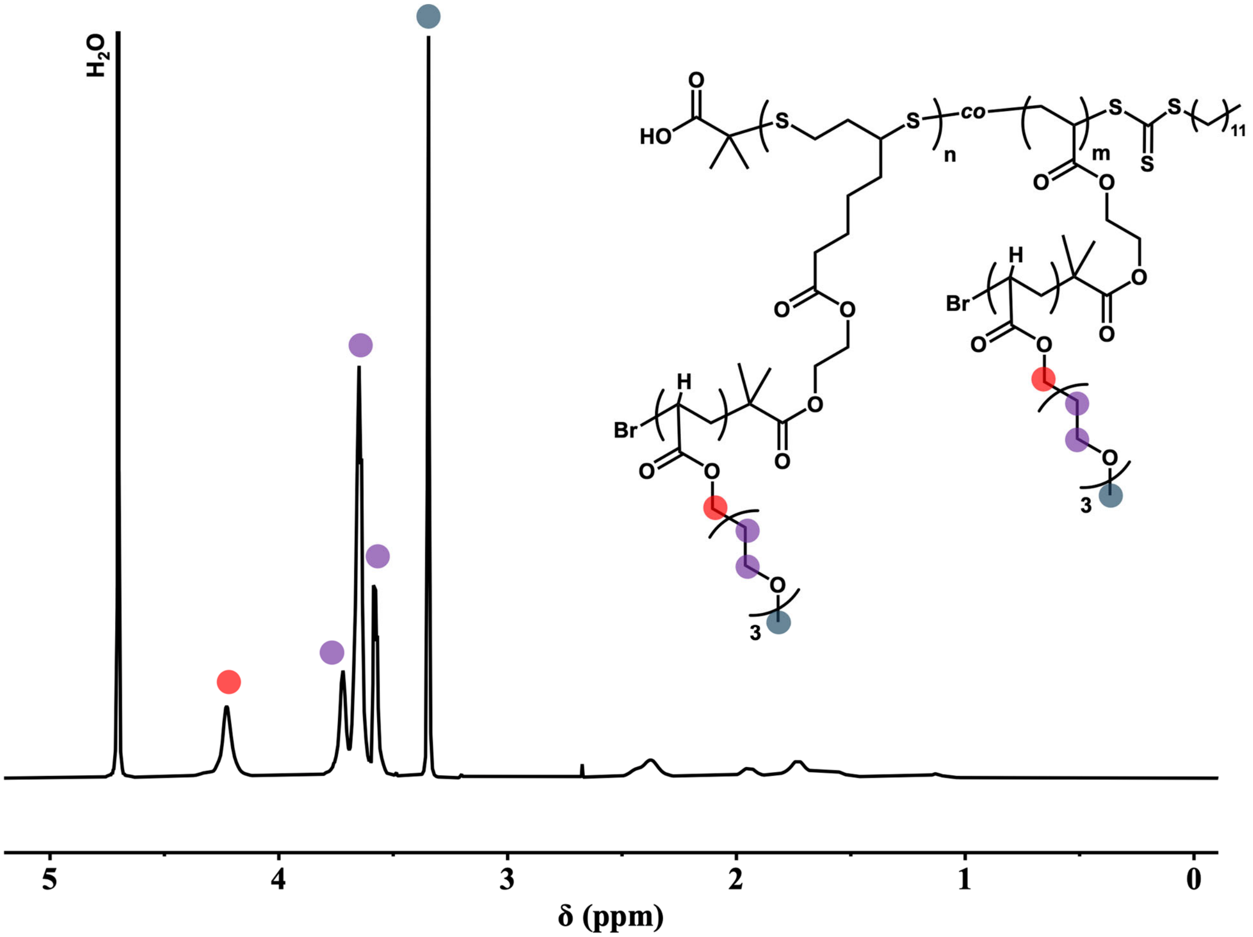

Figure S8. ^{1}H NMR spectrum (500 MHz) of poly(tri(ethylene glycol) methyl ether acrylate) (PTEGA) BB recorded in D_2O.

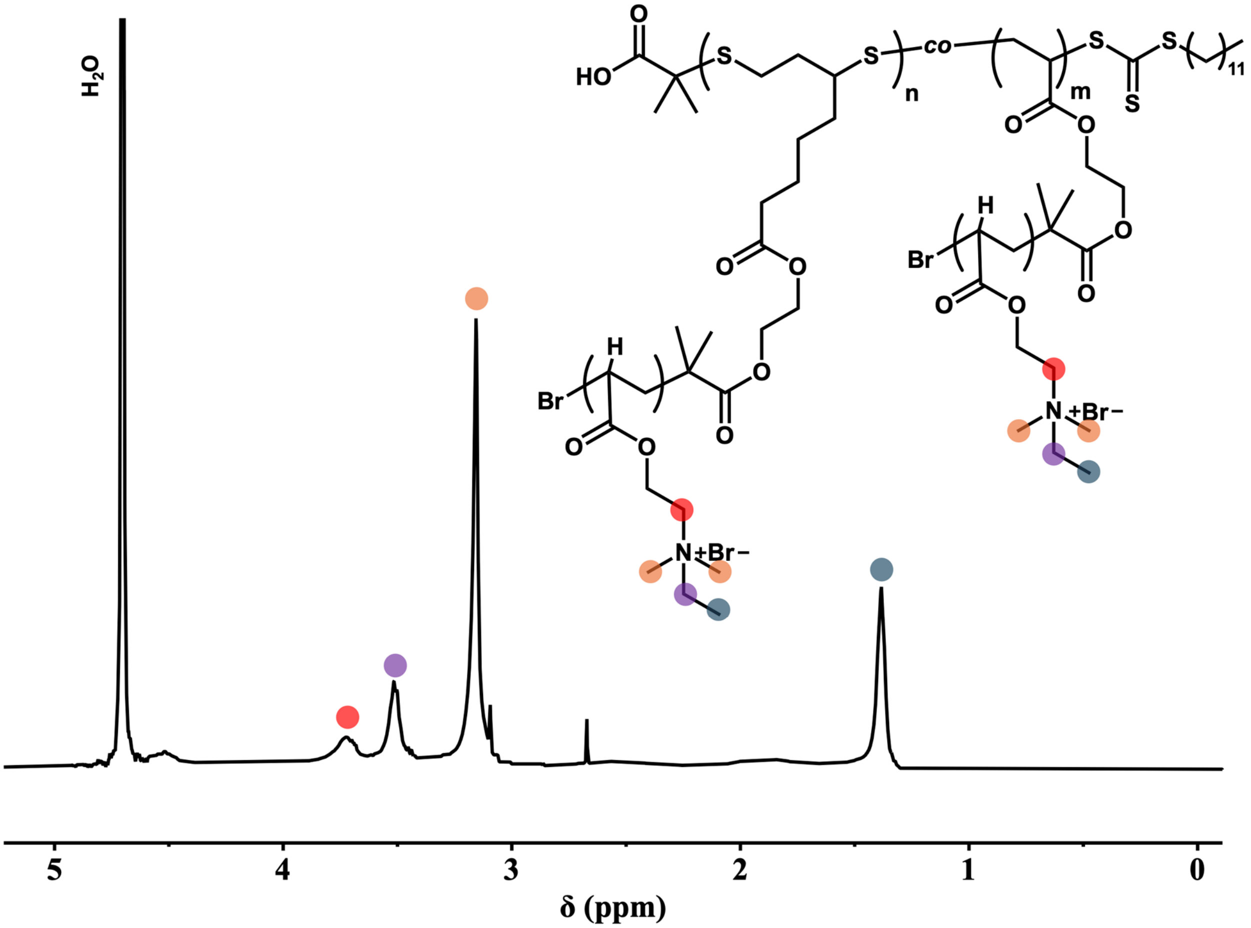

Figure S9. ^{1}H NMR spectrum (500 MHz) of poly(*N*-ethyl-*N,N*-dimethylethylammonium acrylate) (PDMEAA) BB recorded in D_2O.

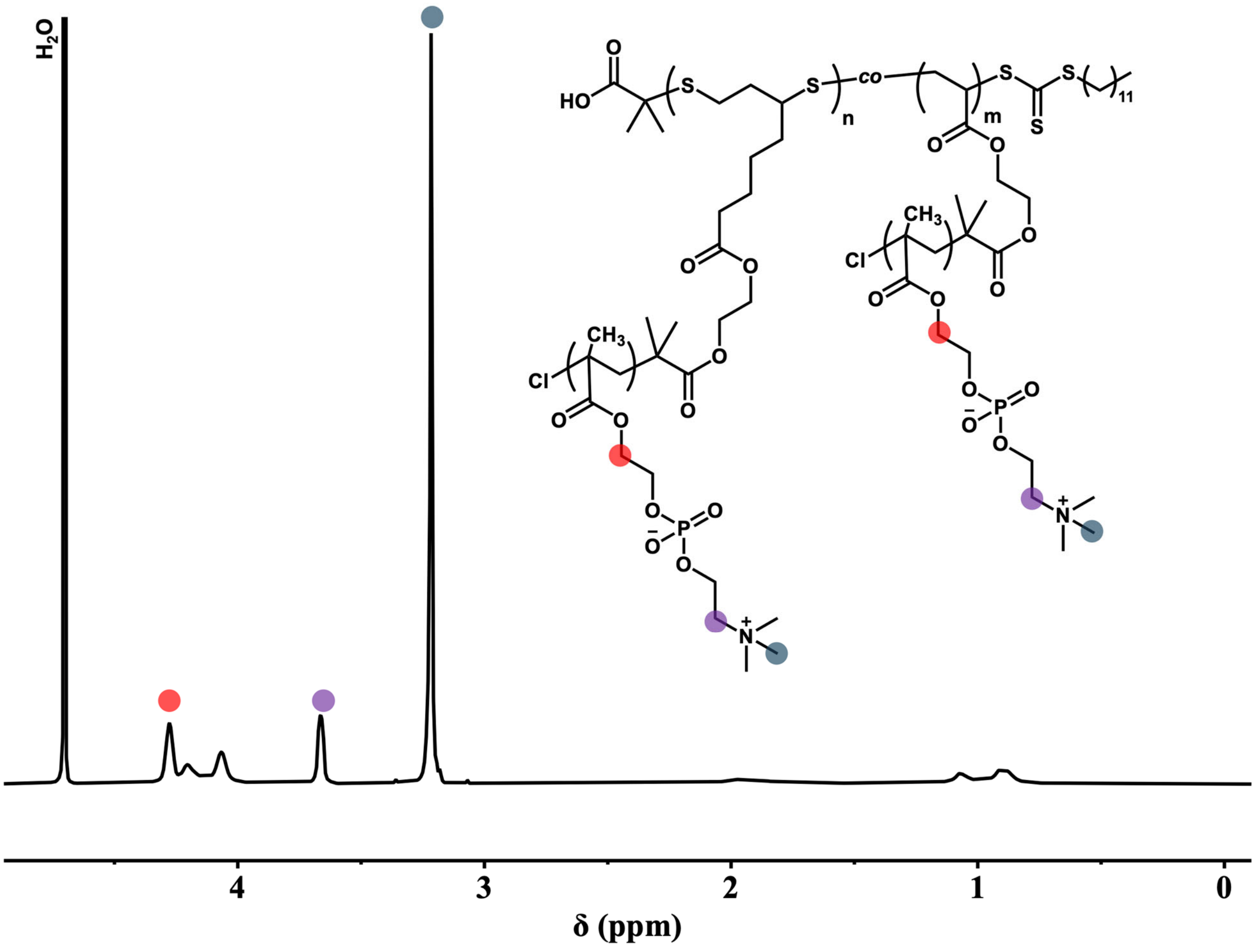

Figure S10. ^{1}H NMR spectrum (500 MHz) of poly(2-methacryloxylethyl phosphorylcholine) (PMPC) BB recorded in D_2O.

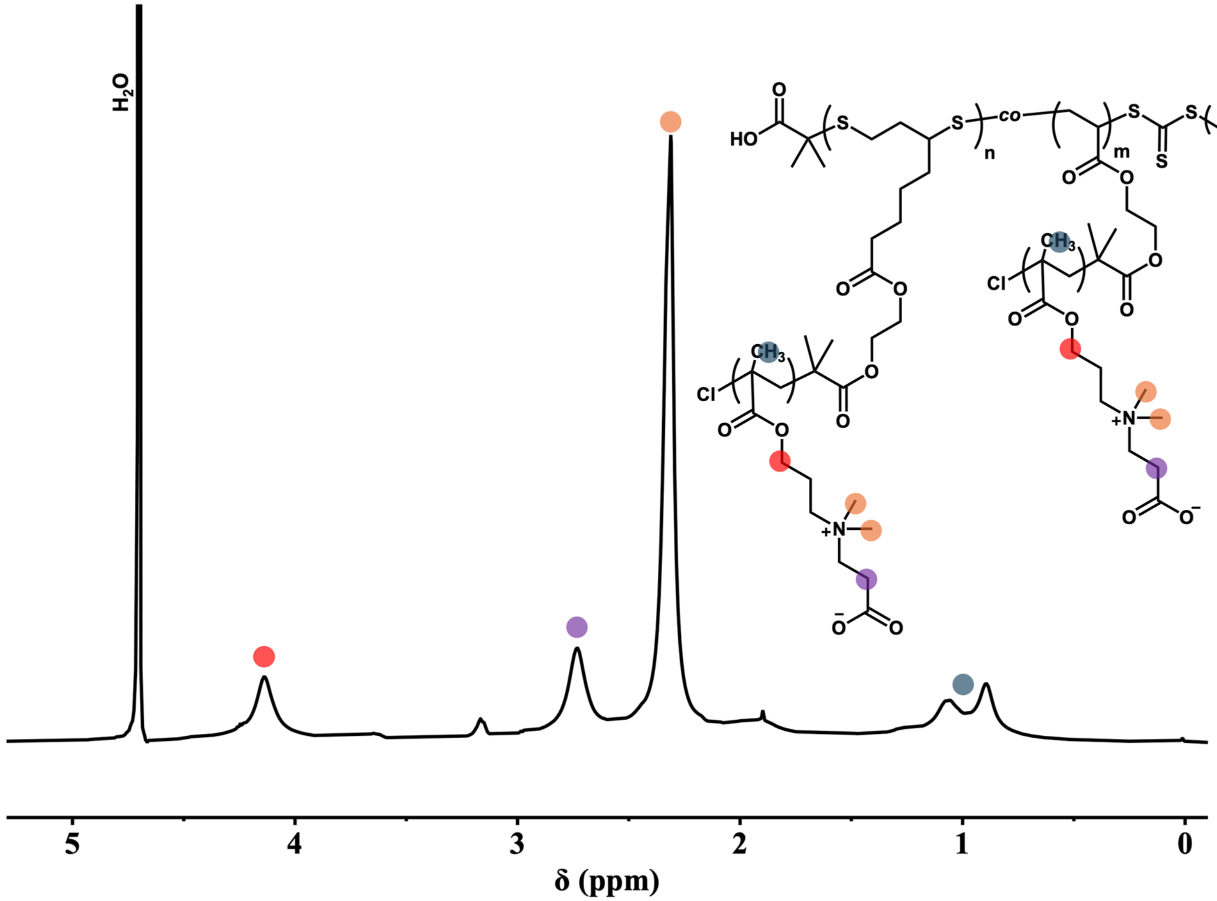

Figure S11. ^{1}H NMR spectrum (500 MHz) of poly(3-[[2-(methacryloyloxy) ethyl]dimethylammonio]propionate) (PCBMA) BB recorded in D_2O.

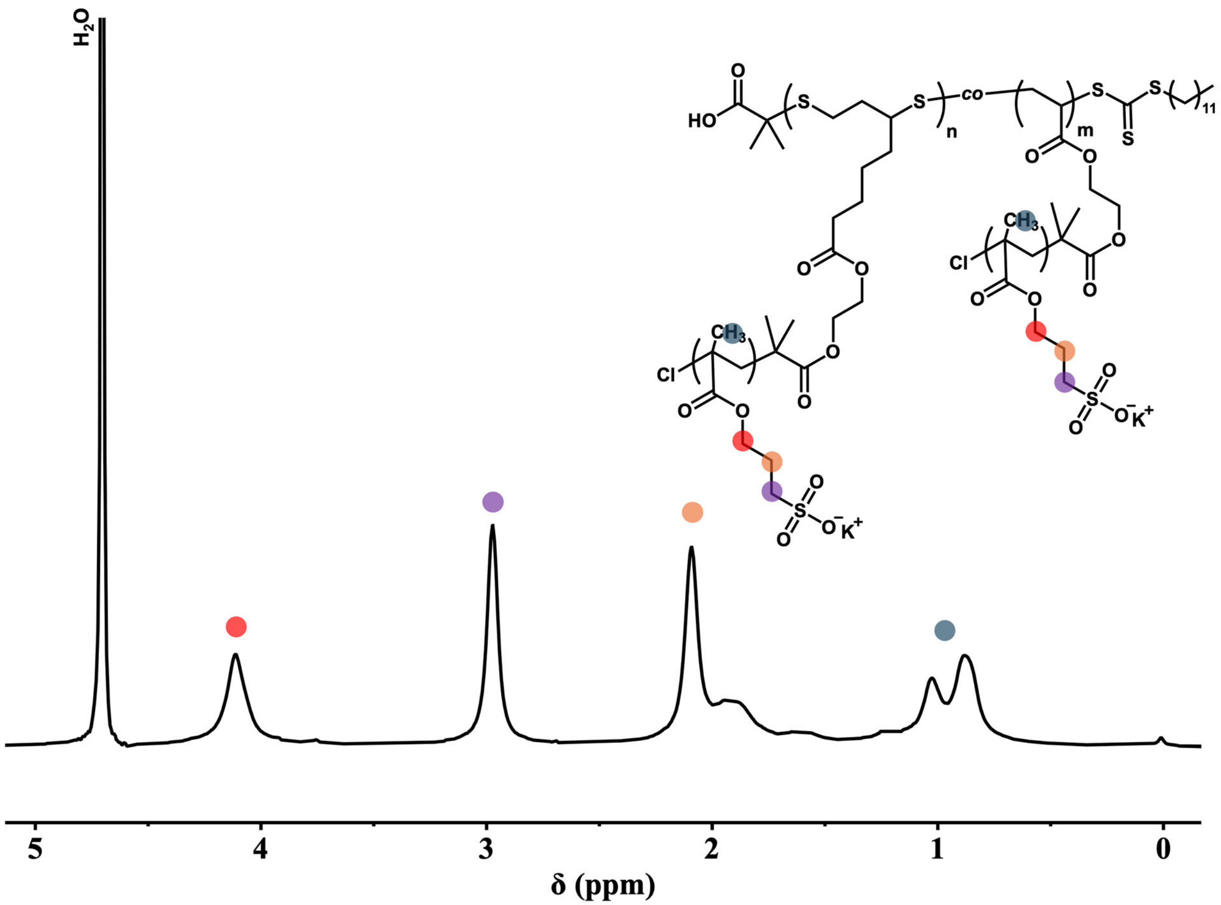

Figure S12. ^{1}H NMR spectrum (500 MHz) of poly(3-sulfopropyl methacrylate) (PSPMA) BB recorded in D_2O.

References

(1) White, M. A.; Johnson, J. A.; Koberstein, J. T.; Turro, N. J. Toward the Syntheses of Universal Ligands for Metal Oxide Surfaces: Controlling Surface Functionality through Click Chemistry. Journal of the American Chemical Society **2006**, 128 (35), 11356–11357. https://doi.org/10.1021/ja064041s.

(2) Lai, J. T.; Filla, D.; Shea, R. Functional Polymers from Novel Carboxyl-Terminated Trithiocarbonates as Highly Efficient RAFT Agents. *Macromolecules* **2002**, *35* (18), 6754–6756. https://doi.org/10.1021/ma020362m.

(3) Grothe, D. C.; Meyer, W.; Janietz, S. Acrylate Functionalized Tetraalkylammonium Salts with Ionic Liquid Properties. *Molecules* **2012**, *17* (6), 6593–6604. https://doi.org/10.3390/molecules17066593.
